# A Schwannoma of the Soft Palate in a Child: Histological and Immunohistochemical Features and Surgical Method

**Published:** 2012

**Authors:** Amin Rahpeyma, Amir Hosein Jafarian, Saeedeh Khajeh Ahmadi, Javad Sarabadani

**Affiliations:** 1 *Department of oral and maxillofacial surgery, Oral and Maxillofacial Diseases Research Center, Faculty of Dentistry, Mashhad University of Medical Sciences, Mashhad, Iran.*; 2 *Department of pathology, Ghaem Hospital, Mashhad University of Medical Sciences, Mashhad, Iran.*; 3 *Department of oral and maxillofacial pathology, Dental Research Center, Faculty of Dentistry, Mashhad University of Medical Sciences, Mashhad, Iran.*; 4 *Department of Oral and Maxillofacial Diseases, Oral and Maxillofacial Disease Research Center, Faculty of Dentistry, Mashhad University of Medical Sciences, Mashhad, Iran*

**Keywords:** Immunohistochemistry, Schwannoma, Soft palate, Surgical procedures

## Abstract

**Introduction:**

Schwannoma, or neurilemmoma, is a benign neoplasm of Schwann cells that is extremely rare in the soft palate. Herein we present a case of a soft palate schwannoma presenting with an ulcerated surface and purplish colour in a 12 year-old girl. This report also introduces a successful surgical technique for coverage of the defect left by surgery.

## Introduction

 A neurilemmoma is a benign, encapsulated tumour that arises from a nerve sheath. One-fourth of extracranial schwannomas occur in the head and neck area. Only 1% of schwannomas present in the oral cavity (1, 2). An oral schwannoma grows slowly and is encapsulated. The most common oral site is the tongue (3) and soft palate involvement is rare. A bilateral schwannoma of the auricular-vestibular nerve is a characteristic feature of neurofibromatosis type II (4, 5) and patients with multiple nerve schwannomas should be evaluated for Von Recklinghaunsen’s disease.

Histologically, neurilemmomas contain two main tissue regions: Antoni A and Antoni B (6). Malignant changes have been reported in long-lasting lesions and conservative surgery is preferred in the treatment of schwannomas (7). Soft palate surgeries can have very important consequences as decreasing the length of the soft plate can cause velopharyngeal incompetence, alteration in speech, and difficulty in swallowing (8). 

The aim of the present study is to report the case of a 12-year-old female patient with an ulcerated mass in her soft palate, and to discuss the clinical picture, histopathology, and immunohistochemistry, as well as the method of surgery and its follow-up for 6 months postoperatively.

## Case report

**Fig 1:**
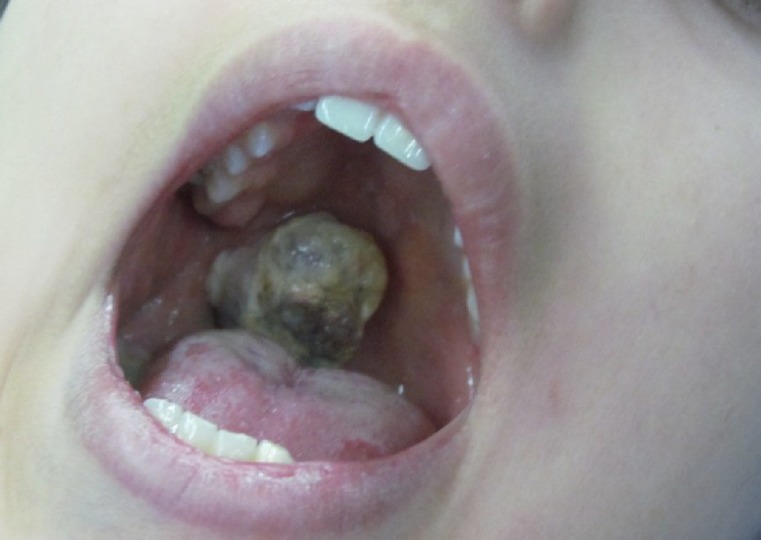
The schwannoma presented as a sessile mass in the right side of the soft palate with an ulcerated surface and purple-yellow discoloration.

A 12-year-old female patient presented without any systemic problems. She had noticed a bulging in her soft palate three months earlier. The patient’s parents reported an increase in the size of the bulge two weeks previously. On intraoral examination, a mass with an ulcerated surface was noted in the soft palate. The lesion extended from the hard palate to near the uvula on the right side, was 3 cm at its largest diameter, and had an ugly appearance. The patient reported no pain, bleeding, or difficulty in swallowing or breathing (Fig. 1).

**Fig 2:**
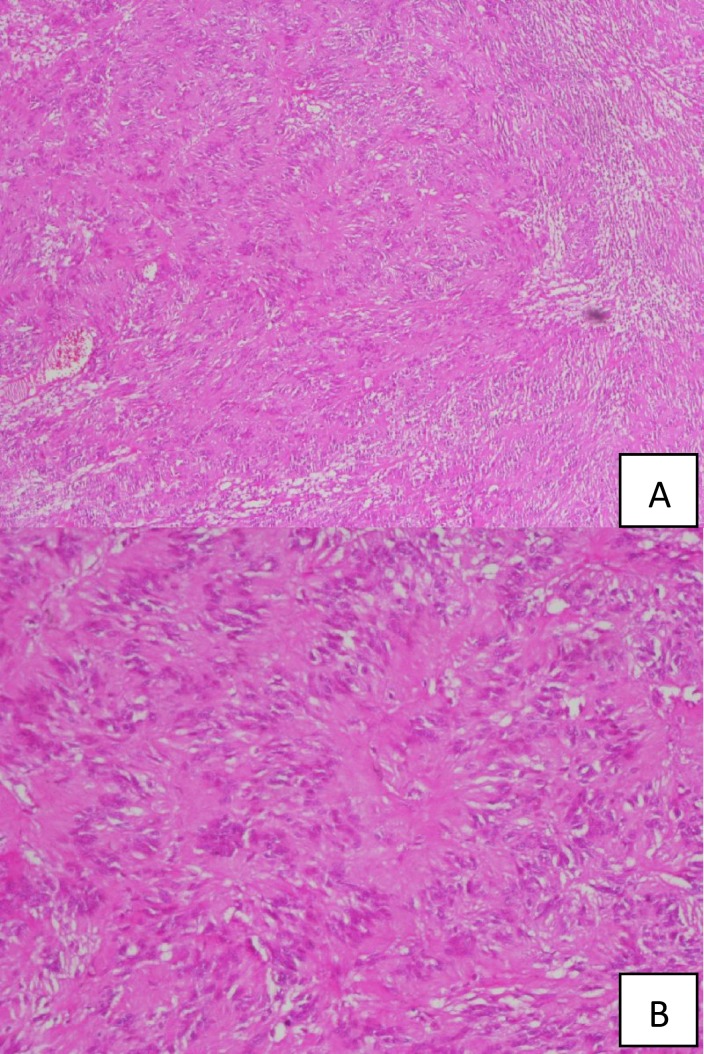
**A-**The lesion contained Antoni type A tissue with Verocay bodies (100×, hematoxilin-eosin). B - (400×, hematoxilin-eosin).

**Fig 3:**
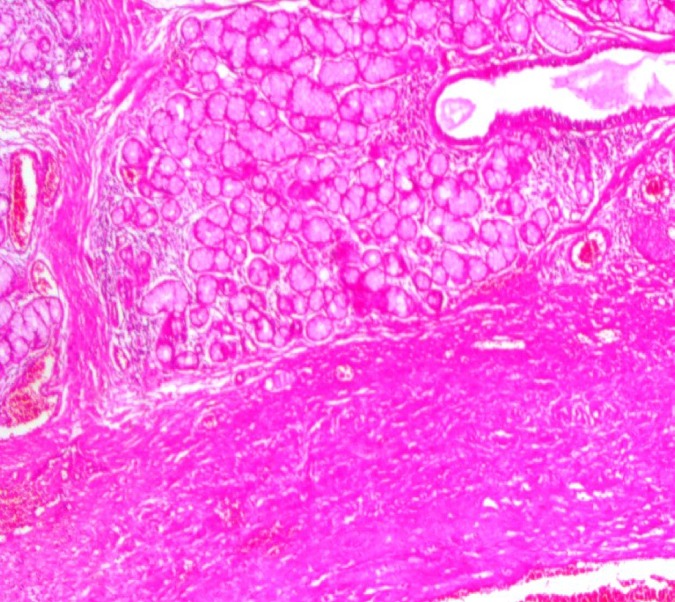
Normal minor salivary glands of the soft palate were visible near the lesion (100× hematoxilin-eosin).

**Fig 4:**
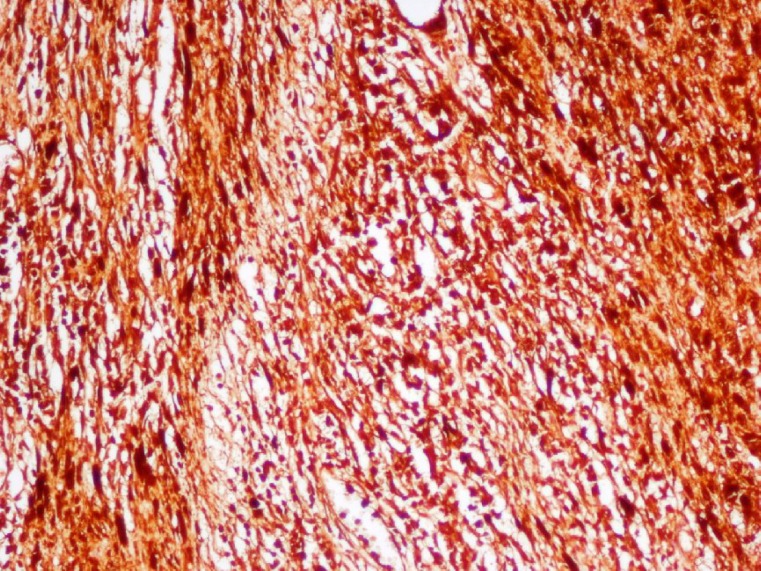
Immunohistochemical staining demonstrated spindle-shaped Schwann cells that were strongly positive for S-100 protein (100×, magnification).

After an incisional biopsy, closure of the wound edges was problematic. A piece of Surgicel^®^ was fixed between the two edges of the tissue and then sutures were placed in normal mucosa far away at the edge of the lesion. Microscopically, the lesion was encapsulated and consisted of Antoni A tissue with Antoni type B regions. The Antoni A tissue included Verocay bodies and was marked by spindle-shaped Schwann cells palisaded around the amorphous eosinophilic area. Numerous blood vessels were observed between these areas (Fig. 2). Adjacent to the lesion, normal salivary glands of the soft palate could be seen (Fig. 3). The results of immunohistochemical staining were strongly positive for S-100 protein (Fig. 4).

**Fig 5:**
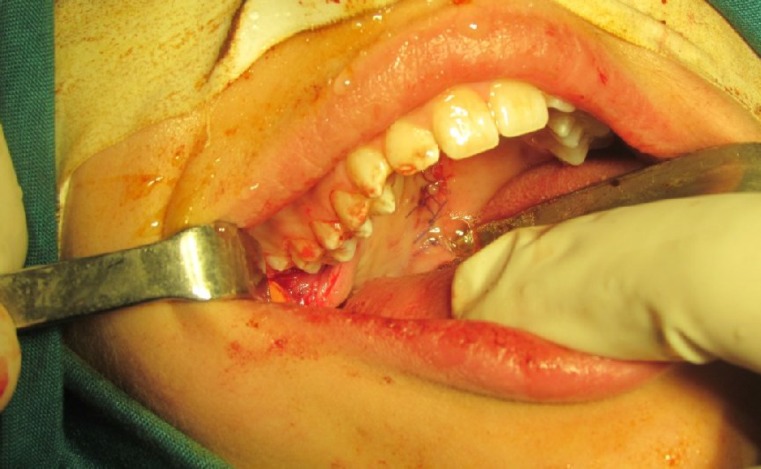
A buccinator myomucosal island pedicle flap was used for reconstruction of the soft palate defect.

According to the clinical features and the histological and immunohistochemical studies, the diagnosis was that the mass was a neurilemmoma. The lesion was removed under general anaesthesia. Macroscopically, the tumour was grossly similar to a haemangioma. Gross inspection of the lesion revealed a purplish, well circumscribed mass, approximately 3 cm x 2 cm x 1 cm in size. The lesion was dissected from the soft palate tissue and the resulting soft palate defect was covered by a posterior-based buccinators myomucosal pedicle flap, while the posterior joint of the buccinators muscle and overlying mucosa were preserved. The base of the flap was separated from the underlying tissue and rotated 180° to cover the defect in the palate (Fig. 5). Secondary surgery was not required for division of the pedicle. After 6 months of follow-up, no evidence of recurrence was detected.

## Discussion

Schwannomas are benign neoplasms that were first described by Verocay in 1908 (9).

Neurilemmomas have two main regions: Antoni A and Antoni B. Antoni A regions consist of fascicles of spindle-shaped Schwann cells arranged around an eosinophilic area surrounded by a palisade of spindle cells. The cells of the Verocay bodies are oval or linear in shape. Antoni B regions consist of fewer Schwann cells spread in loose, myxomatous stroma that lack the organoid Verocay bodies (6). In this case, inspection revealed that both Antoni A and Antoni B tissue was present, as well as numerous blood vessels and hyalinized vessels. This may explain why the lesion was clinically similar to a hemangioma.

Approximately half of the cases of head and neck schwannomas present in the oral cavity (20% to 58%). Only 1% of this type of tumour occurs in the jaw (10). A lesion of the oral cavity usually occurs in soft tissue; the tongue is the most common location. Oral schwannomas exhibit a slow growth pattern and are encapsulated. A schwannoma tumour rarely occurs in the soft palate. Neurilemmoma is typically diagnosed between the ages of 25 and 55 (3) and children are rarely affected. Until 2010, only a few cases of lingual schwannomas in children had been reported (9). The present patient was a 12-year-old girl with a three-month history of a schwannoma in her soft palate.

Although these tumours are of nervous origin, the patient experiences no pain, but pressure from the tumour on an adjacent nerve may present as paraesthesia. There was no pain or paraesthesia in the present case. A neurilemmoma presents as a submucosal nodule with normal surface coloration. The present lesion was a painless mass that was purple in coloration and had an ulcerated surface. Schwannomas usually do not become as large as 2 cm^3^, but in this case the patient’s tumour had grown to roughly 6 cm^3^. 

Malignant changes in untreated neurilemommas have been reported only rarely (1). Conservative surgical excision is the treatment of choice for schwannomas (7), but surgical removal of vestibular schwannomas in patients with neurofibromatosis type II is difficult. Soft palate surgeries can affect soft palate functions as decreasing the length of the soft plate can cause velopharyngeal incompetency, alteration in speech and difficulty in swallowing (8). We resected the lesion and simultaneously reconstructed the defect left by the surgery with a buccinator myomucosal pedicle flap (11). Originally described as a cheek flap by Mukherji, who used it in primary palatoplasty of short palates (12), this flap is a posteriorly based, random pattern flap with its base situated near the retromolar trigone. The distal end of the flap is the retromolar trigone, while the anterior border is located 1 cm posterior to the oral commissure and the superior border is restricted by the stenson papilla. A pivot point in the posterior border of the flap enables it to cover soft plate defects even in dentated patients.

The clinical differential diagnosis for soft tissue masses in the submucosa are: traumatic neuroma, neurofibroma, mucosal neuroma, haemangioma, mucocele, fibromas, lipomas, and salivary gland tumours (13). The clinical differential diagnosis of the present case was haemangioma, based on the surface colour. Mucosal haemangiomas present typically as painless, soft, and self-limited, with a red or blue surface colour. The lesions are lobulated and blanch with finger pressure. A diascopy test on the patient was negative. Intraoral haemangiomas typically appear in patients who are older than patients who present with similar lesions in other places. There is a strong tendency for haemangiomas to present in males, but the present patient was a 12-year-old girl.

## Conclusion

Since soft palate surgeries can affect swallowing and speech, we propose a surgical technique which leaves less of a defect in the palate.
